# Tissue Expression of Atrial and Ventricular Myosin Light Chains in the Mechanism of Adaptation to Oxidative Stress

**DOI:** 10.3390/ijms21218384

**Published:** 2020-11-09

**Authors:** Marta Banaszkiewicz, Anna Krzywonos-Zawadzka, Agnieszka Olejnik, Iwona Bil-Lula

**Affiliations:** Division of Clinical Chemistry and Laboratory Hematology, Department of Medical Laboratory Diagnostics, Faculty of Pharmacy, Wroclaw Medical University, Borowska 211 A, 50-556 Wroclaw, Poland; marta.banaszkiewicz@umed.wroc.pl (M.B.); anna.krzywonos-zawadzka@umed.wroc.pl (A.K.-Z.); agnieszka.olejnik@student.umed.wroc.pl (A.O.)

**Keywords:** ALC1, VLC1, ischemia, reperfusion, adaptation, injury, heart mechanical function

## Abstract

Ischemia/reperfusion (I/R) injury induces post-translational modifications of myosin light chains (MLCs), increasing their susceptibility to degradation by matrix metalloproteinase 2 (MMP-2). This results in the degradation of ventricular light chains (VLC1) in heart ventricles. The aim of the study was to investigate changes in MLCs content in the mechanism of adaptation to oxidative stress during I/R. Rat hearts, perfused using the Langendorff method, were subjected to I/R. The control group was maintained in oxygen conditions. Lactate dehydrogenase (LDH) activity and reactive oxygen/nitrogen species (ROS/RNS) content were measured in coronary effluents. Atrial light chains (ALC1) and ventricular light chains (VLC1) gene expression were examined using RQ-PCR. ALC1 and VLC1 protein content were measured using ELISA tests. MMP-2 activity was assessed by zymography. LDH activity as well as ROS/RNS content in coronary effluents was higher in the I/R group (*p* = 0.01, *p* = 0.04, respectively), confirming heart injury due to increased oxidative stress. MMP-2 activity in heart homogenates was also higher in the I/R group (*p* = 0.04). ALC1 gene expression and protein synthesis were significantly increased in I/R ventricles (*p* < 0.01, 0.04, respectively). VLC1 content in coronary effluents was increased in the I/R group (*p* = 0.02), confirming the increased degradation of VLC1 by MMP-2 and probably an adaptive production of ALC1 during I/R. This mechanism of adaptation to oxidative stress led to improved heart mechanical function.

## 1. Introduction

The restoration of blood flow to pre-ischemic myocardium leads to ischemia–reperfusion injury (IRI), which subsequently results in deterioration of cardiac function [1,2,3,4,5,6]. Numerous clinical manifestations of IRI including myocardial necrosis, arrhythmia, and endothelial and microvascular dysfunction are observed [5]. One of the most important mechanisms underlying IRI is a direct damage to the contractile machinery and sarcomeric proteins degradation [4,5,6,7]. IRI results in triggering pathways that lead to post-translational modifications—nitration, nitrosylation and phosphorylation of myosin light chains 1 and 2 (MLCs1 and MLCs2, respectively) [6,7,8,9]. These post-translational modifications make MLCs and other proteins more susceptible to degradation by matrix metalloproteinase 2 (MMP-2) [10,11]. Moreover, increased oxidative stress during IRI induced MMP-2 activation in the myocardium [12,13,14,15,16]. This proves that MMP-2 plays an important role in contractile dysfunction and heart damage [1,2,12]. The degradation of ventricular light chains 1 (VLC1) by MMP-2 in heart ventricles in chronic heart disease causes the re-expression of atrial–fetal light chains 1 (ALC1/FLC1) [17,18,19,20,21,22]. ALC1 is an alkali myosin light chain 1, which appears only in heart atria in healthy adults, while it can be found both in the atria and ventricles in fetus—it then appears as FLC1 [23,24,25,26,27,28,29,30]. Its expression in the ventricles gradually disappears during individual development, and ALC1 is completely replaced by VLC1 in adult heart ventricles [17,23]. People with congenital heart diseases such as hypertrophic or dilated cardiomyopathy, valvular heart disease, and tetralogy of Fallot [27,28,31] are the exceptions, since ALC1 is expressed in their ventricles during adult life [25,26,32]. ALC1 re-expression in the ventricles is associated with the compensation of heart failure and improvement of its contractility [17,18,19,20,21,31,32,33]. It has been shown that even a low level of ALC1 reconstitution in the heart ventricles significantly improves the functional state of myosin and thus myocardial contractility [23].

The main aim of this study was to investigate whether ALC1 expression was increased in rat heart ventricles as an adaptive mechanism to ischemia–reperfusion injury. We explored changes in gene expression and concentration of ALC1 and VLC1 proteins in isolated rat heart ventricles. We also measured reactive oxygen/nitrogen species (ROS/RNS) and MMP-2 content, as well as MMP-2 activity in hearts as indicators of oxidative stress. We analyzed the hemodynamic parameters of perfused hearts and examined the contractility of isolated myocytes to assess the impact of VLC1 degradation on heart function.

## 2. Results

### 2.1. An Influence of Ischemia/Reperfusion on Heart Mechanical Function and Injury

Heart rate (HR), coronary flow (CF), and recovery of heart mechanical function (RPP) (rate pressure product at 77 min versus 25 min of perfusion—the product of HR and left ventricular developed pressure (LVDP)—systolic minus diastolic ventricular pressure) were significantly decreased in hearts subjected to global no-flow ischemia and reperfusion in comparison to aerobic control (Table 1).

**Figure 1:**
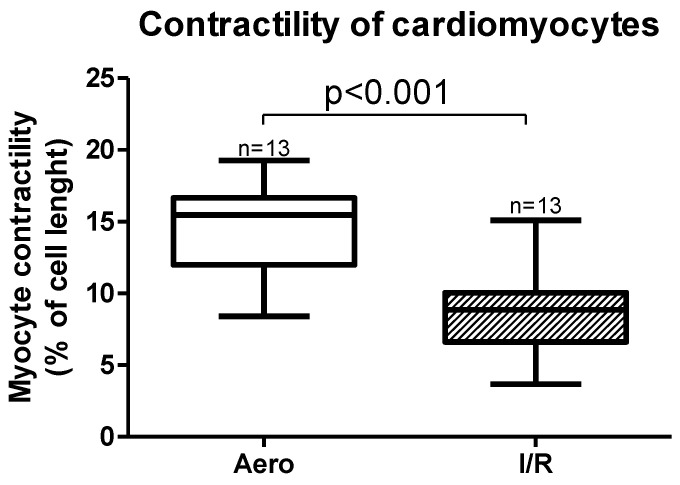
Myocytes contractility after ischemia and in aerobic conditions. Aero—aerobic control group, I/R—ischemia/reperfusion group; all data are expressed as mean ± SEM.

Cardiomyocytes contractility, expressed as a peak shortening (% of cell length), was also significantly decreased (*p* < 0.001, *n* = 13) after ischemia and reperfusion (Figure 1).

**Figure 2:**
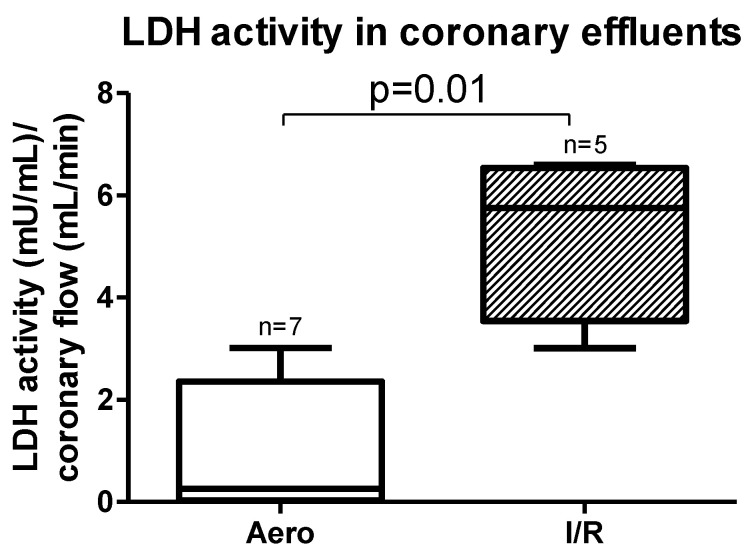
Lactate dehydrogenase (LDH) activity in coronary effluent after ischemia/reperfusion (I/R) and in aerobic conditions; all data are expressed as mean ± SEM.

Lactate dehydrogenase (LDH) activity measured in coronary effluents was significantly increased in the I/R group in comparison to the Aero group (*p* = 0.01, *n* = 5–7) (Figure 2), confirming cardiomyocyte damage during I/R.

### 2.2. Oxidative Stress during Ischemia/Reperfusion Injury

**Figure 3:**
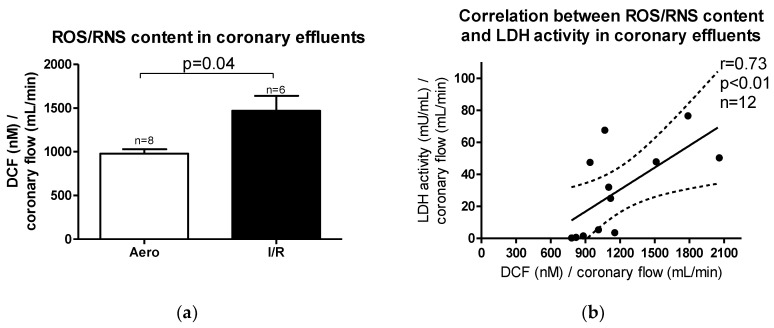
(**a**) Reactive oxygen/nitrogen species (ROS/RNS) content in coronary effluents; (**b**) Correlation between ROS/RNS content and lactate dehydrogenase (LDH) activity in coronary effluents. DCF-2′,7′–dichlorodihydrofluorescein; all data are expressed as mean ± SEM.

Reactive oxygen/nitrogen species (ROS/RNS) content in coronary effluents was increased after ischemia/reperfusion (I/R) in relation to aerobic control (*p* = 0.04, *n* = 6–8) (Figure 3a). A positive correlation between ROS/RNS and LDH activity in coronary effluents (*p* < 0.01, r = 0.73) was observed (Figure 3b), suggesting that heart injury was strongly associated with ROS/RNS production during oxidative stress.

### 2.3. MMP-2 Synthesis and Activity in Heart Homogenates Subjected to Ischemia/Reperfusion Injury

**Figure 4:**
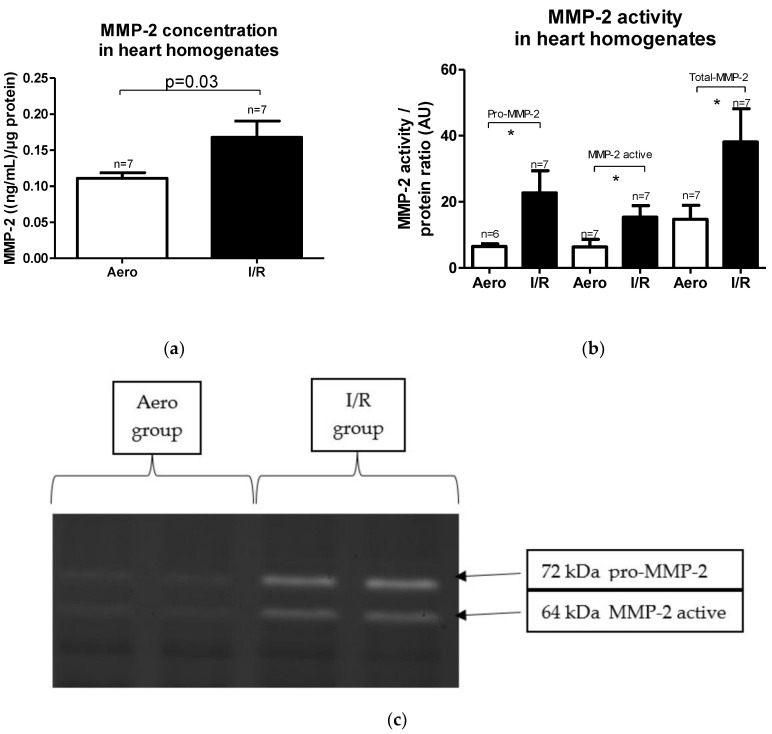
(**a**) Matrix metalloproteinase 2 (MMP-2) concentration in heart homogenates normalized to protein concentration in each sample; (**b**) pro/active/Total-MMP-2 activity in heart homogenates; (**c**) zymograms presenting MMP-2 activity, * *p* < 0.05 vs. aerobic control; all data are expressed as mean ± SEM. AU—arbitrary units.

MMP-2 concentration in heart homogenates as well as its activity were increased in the I/R group in comparison to the Aero group (*p* = 0.03, pro-MMP-2: *p* = 0.04, MMP-2 active: *p* = 0.03, Total-MMP-2: *p* = 0.04, respectively, *n* = 7) (Figure 4a–c).

**Figure 5:**
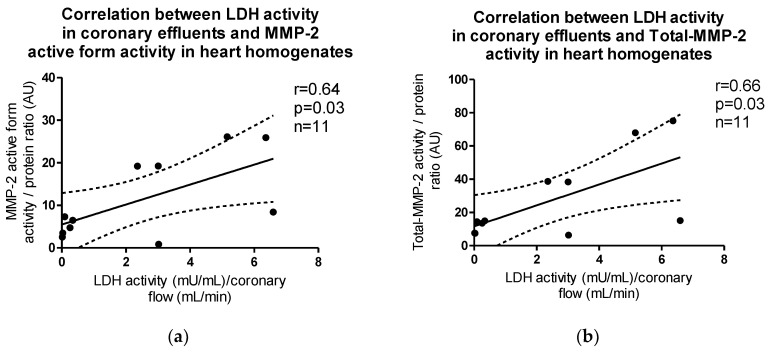
Correlations between LDH activity in coronary effluents and (**a**) MMP-2 active form, (**b**) Total-MMP-2 activity in myocytes.

The activity of both active and total forms of MMP-2 in heart homogenates correlated positively with LDH activity in coronary effluents (*p* = 0.03, r = 0.64; *p* = 0.03, r = 0.66, respectively) (Figure 5a,b).

### 2.4. MYL4, MYL3 mRNA Expression and ALC1, VLC1 Concentrations in Rat Hearts

**Figure 6:**
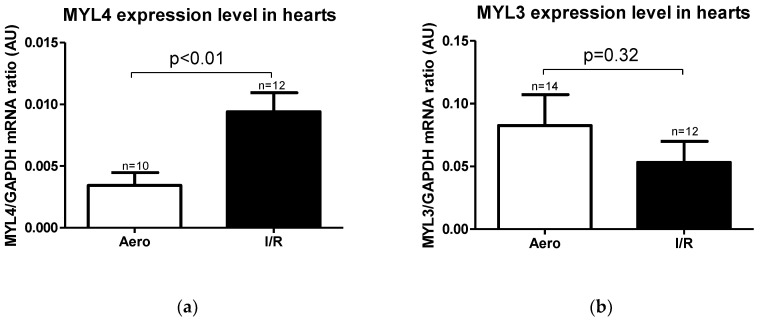
(**a**) Myosin light chain 4 (*MYL4*) and (**b**) Myosin light chain 3 (*MYL3*) mRNA expression level normalized to the *GAPDH* gene. GAPDH—glyceraldehyde 3-phosphate dehydrogenase; all data are expressed as mean ± SEM.

Expression level of *MYL4* gene (coding ALC1 protein) was significantly higher in the I/R group in relation to the Aero group (*p* < 0.01, *n* = 10–12), while the *MYL3* gene (coding the VLC1 protein) expression level result was not significant (*p* = 0.32, *n* = 12–14) (Figure 6a,b).

**Figure 7:**
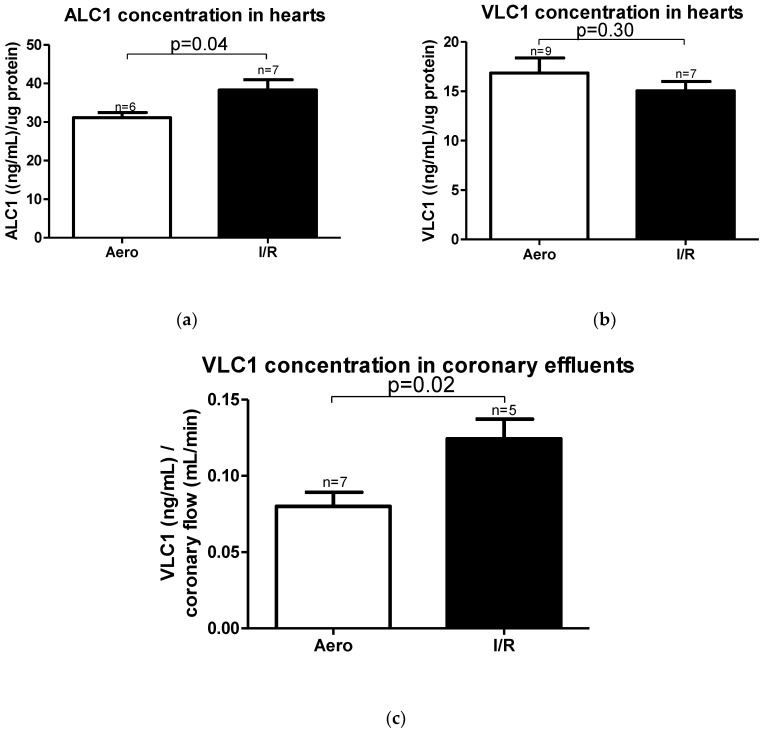
(**a**) Atrial light chain 1 (ALC1) and (**b**) ventricular light chain 1 (VLC1) concentrations in heart homogenates measured using ELISA tests and normalized to the content of protein in each sample; (**c**) VLC1 concentration in coronary effluents normalized to coronary flow; all data are expressed as mean ± SEM.

According to mRNA expression, the ALC1 protein content in heart homogenates was significantly increased in the I/R group (*p* = 0.04, *n* = 6–7) (Figure 7a), and no changes in VLC1 concentration in heart homogenates were observed (*p* = 0.30, *n* = 7–9) (Figure 7b). However, VLC1 content in coronary effluents was substantially increased in the I/R group in comparison to Aero group (*p* = 0.02, *n* = 5–7), confirming that VLC1 was released into extracellular space (Figure 7c).

**Figure 8:**
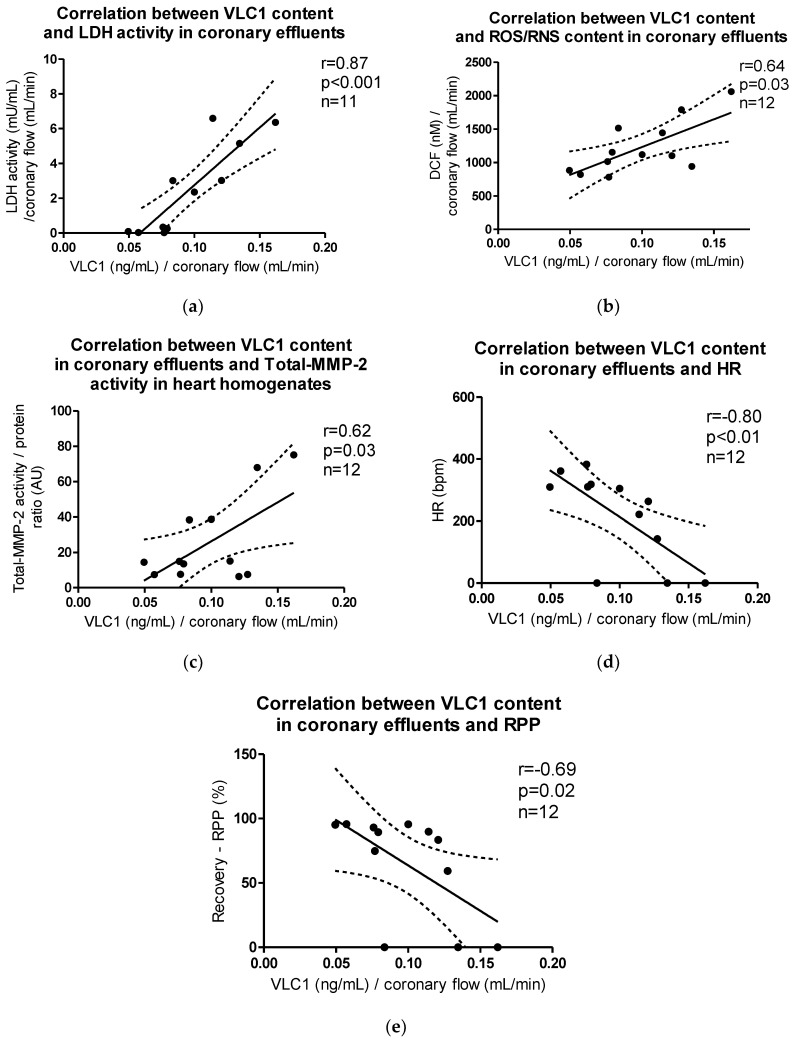
Correlations between VLC1 content in coronary effluents and (**a**) LDH activity, (**b**) ROS/RNS content, (**c**) Total-MMP-2 activity in myocytes, (**d**) heart rate (HR), (**e**) recovery of heart mechanical function (RPP).

VLC1 content in coronary effluents correlated positively with LDH activity (*p* < 0.001, r = 0.87), ROS/RNS content in coronary effluents (*p* = 0.03, r = 0.64), and Total-MMP-2 activity in heart homogenates (*p* = 0.03, r = 0.61), while the negative correlation between VLC1 content in coronary effluents and HR (*p* < 0.01, r = −0.80) and recovery of rate pressure product (RPP) (*p* = 0.02, r = −0.69) was observed (Figure 8a–e).

## 3. Discussion

In this study, we aimed to explore changes in heart contractile machinery due to ischemia–reperfusion injury. We showed that increased ROS/RNS release into coronary effluents and MMP-2 content and activity in heart homogenates affected VLC1 degradation in heart ventricles and increased it. Myocytes contractility as well as hemodynamic parameters measured during heart perfusion were decreased after I/R. We revealed that ALC1 gene expression and content were increased in heart ventricles, which may constitute a compensatory mechanism to I/R conditions and increased oxidative stress.

Myosin molecule consists of two heavy chains, myosin heavy chain α (MHC-α) and myosin heavy chain β (MHC-β), and two pairs of light chains: regulatory (RLC/MLC2) and essential (ELC/MLC1), divided into atrial (ALC1) and ventricular (VLC1) [13,17,31,34,35,36]. Since MHC have actin and ATP binding sites and present the molecular motor of muscle contraction [26,27,28,30,32], ELCs (MLCs1) are primarily responsible for maintaining MHC conformation and regulating contraction strength and speed [1,21,37,38]. In the current research, we showed that VLCs1 were degraded and released into extracellular space due to I/R-induced oxidative stress, as evidenced by the decreased heart level of VLC1 and its release into coronary effluents. The release of VLC1 was associated with heart damage and an increased activation of MMP-2, which was confirmed by the positive correlation of VLC1 concentration and LDH activity (marker of tissue injury) in coronary effluents, as well as an activity of MMP-2 in heart homogenates. In addition, a positive correlation of VLC1 and ROS/RNS content in coronary effluents as well as Total-MMP-2 activity suggested an induction of MMP-2 activity due to oxidative stress. Furthermore, the increased activity of MMP-2 and enlarged degradation of contractile proteins followed by their release into the extracellular space led to decreased HR, CF, and RPP, indicating that the degradation of contractile proteins led to a decrease in heart function. The negative correlation between VLC1 content in coronary effluents and heart rate (HR) may indicate that the degradation of VLC1 and its release into extracellular space caused a deterioration of myocytes contractility, which affected heart rate. Heart rate is the speed of heartbeat measured by the number of contractions of the heart per minute. Contractions decreased, which was shown not only in decreased HR but also in decreased myocytes contractility.

In many previous studies, MMP-2 had been shown to be involved in the degradation of contractile proteins [14,38,39]. In this study, we also showed that an increased production of reactive oxygen and nitrogen species during IRI was accompanied by an increased concentration and activity of all three forms of MMP-2. Moreover, the contractility of cardiomyocytes was significantly reduced in the I/R group compared to the control. This was associated to increased MMP-2 activity, which degraded contractile proteins and thus reduced contractility. Wang et al. (2002) proved, as we did, that in both human and rat cardiomyocytes, MMP-2 expression was increased after the ischemia [12]. The same conclusions were also reached by Lin et al. (2014), who examined the activity of MMP-2 in intact isolated hearts of adult rats and observed an increase in MMP-2 activity in a very short time after ischemia [14]. Fert-Bober et al. (2007) showed an increased MMP-2 activity in coronary effluents after I/R. This was accompanied by cardiac disorder, which they were trying to prevent by inhibiting MMP-2. They revealed that the inhibition of MMP-2 activity had a cardioprotective effect, as it reduced heart dysfunction and endothelial damage [40]. Lin et al. (2014) also inhibited MMP-2 activity using MMP-2 siRNA and revealed that VLC1 degradation was decreased after MMP-2 inhibition [14].

Our results show that I/R of heart tissue led to a decreased content of VLC1 and an increased tissue expression of ALC1 in ventricular cardiomyocytes in comparison to the Aero group. We found no correlation of ALC1 content in heart homogenates with MMP-2 activity in heart homogenates and LDH activity in coronary effluents. ALC1 content in coronary effluents showed no differences between the aerobic control group and I/R group. Those results may mean that ALC1 did not constitute a target for degradation by MMP-2 and did not contribute to heart damage. It rather might be induced by oxidative stress in order to prevent any further deterioration of cardiac function. The increased expression of ALC1 in heart ventricles was considered as an adaptive mechanism to I/R conditions in cardiac hypertrophy in the previous studies [21,29]. ALC1 is shorter in length than VLC1, and it modulates the speed of myosin cycles—the amount of interactions between ALC1 and actin is reduced, leading to the improvement of heart function [19,33]. ALC1 imposes a lower molecular load on the myosin bridge and binds less to actin, which results in improved contractility [17,21,27]. Since in this study, we showed an increased content of ALC1 in hearts subjected to ischemia and reperfusion, despite an enhanced activity of MMP-2, an increased tissue injury, and the release of VLC1, it can result from ALC1 resistance to MMP-2 related degradation or induce tissue production to marinate the proper heart function; however, this needs further investigation.

In this research, we showed an increased tissue content of ALC1 and decreased content of VLC1 in an ischemia–reperfusion injury model. We revealed an increased amount of ALC1 already at the level of gene expression in rat heart cardiomyocytes after I/R. This proportion was also maintained at the stage of protein synthesis. Arrell et al. (2001) and Morano et al. (1996) showed that a small amount of ALC1 appeared in the ventricles of adult people with chronic cardiovascular disease and hypertrophic, dilated cardiomyopathy, similarly to our I/R mechanism, which increased the maximal shortening velocity, rate of tension redevelopment, and isometric force generation [17,20]. Khalina et al. (2005) explored the effect of recombinant ALC1 on human ventricular myosin activity, and they showed that its function was significantly improved: the actin-activated ATPase activity of myosin increased, which highly contributed to heart function compensation [23]. Petzhold et al. (2011) and Woischwill et al. (2005) presented studies in which they proved that the replacement of endogenous VLC1 by human ALC1 in adult rat cardiomyocytes and the transgenic overexpression of mouse ALC1 in a mouse’s heart ventricle increased cardiac contractility and myosin motor properties [22,32]. Fewell et al. (1998) also presented a study on transgenic mice in which ALC1 was present in the heart ventricles [31]. They showed that the heart did not undergo hypertrophy, as could be expected, but there was a significant change in its motor function. All the above confirmed our hypothesis that VLC1/ALC1 dysproportion, both at the level of mRNA and protein synthesis, could be a compensatory response designed to maintain cardiac function in altered conditions. However, Morano et al. (1999) suggested that the level of ALC1 expression in the ventricles of injured hearts may be too low to properly balance the reduced systolic function, which was also observed in rat hearts ventricles that we examined—significant damage to the heart was still visible [27]. This created the foundation for further studies on the regulation of ALC1 expression by the genetic transfer of ALC1 cDNA into the cardiomyocyte or by upregulation of ALC1 gene transcription [27].

However, there are also reports showing that the mechanism of compensation based on the replacement of degraded VLC1 by ALC1 occurs only in human heart, while rodents present a compensatory mechanism based on change in the expression of MHC [13,17,18,19,29,41,42]. However, as presented above, our results confirmed that the VLC1/ALC1 replacement mechanism occurs also in rat heart. For this reason, the subject requires further study. It could be possible that rodents present a double compensatory mechanism.

## 4. Materials and Methods

This investigation conforms to the Guide to the Care and Use of Experimental Animals published by the Polish Ministry of Science and Higher Education and was approved by the local Ethics Committee for Experiments on Animals at the Ludwik Hirszfeld Institute of Immunology and Experimental Therapy, Polish Academy of Sciences, Wroclaw, Poland (Resolution 14/2016 of 20 April 2016).

### 4.1. Protocol of Isolated Heart Perfusion According to Langendorff Method and of Global Ischemia and Reperfusion of Isolated Rat Hearts

**Figure 9:**
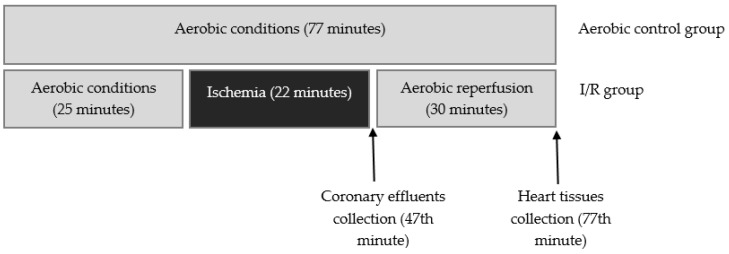
Experimental protocol for I/R group and aerobic control.

Male Wistar rats (weighing 300–350 g) were used in these experiments as a surrogate model for analysis of heart protection. Rats were treated with buprenorfin (0.05 mg/kg, i.p.), anaesthetized with sodium pentobarbital (40 mg/kg, i.p.), and hearts were rapidly excised. Immediately after removal, spontaneously beating hearts were rinsed by immersion in ice-cold Krebs–Henseleit Buffer containing 118 mmol/L NaCl, 4.7 mmol/L KCl, 1.2 mmol/L KH_2_PO_4_, 1.2 mmol/L MgSO_4_, 3.0 mmol/L CaCl_2_, 25 mmol/L NaHCO_3_, 11 mmol/L glucose, and 0.5 mmol/L ethylenediaminetetraacetic acid (EDTA), pH 7.4. Next, hearts were suspended on a blunt end needle of the Langendorff system (EMKA Technologies, Paris, France) with aorta and maintained at 37 °C. Hearts were perfused at a constant pressure of 60 mm Hg with Krebs–Henseleit Buffer at pH 7.4, at 37 °C and gassed continuously with 5% carbogen. After aerobic stabilization (25 min), hearts were subjected to global, no-flow ischemia (22 min, by closing the aortic inflow line) and aerobic reperfusion (30 min). Control hearts were perfused aerobically for 77 min. CF, HR, and LVDP were defined as hemodynamic end points of cardioprotection. The cardiac mechanical function was expressed as the recovery of RPP. Coronary effluents for biochemical tests were collected at the beginning of reperfusion (47 min) to achieve the constant volume (15 mL) (Figure 9). Next, atria were cut off from the isolated hearts, and ventricles were immediately immersed in liquid nitrogen and stored at −80 °C until further analysis.

### 4.2. Protocol of Heart Perfusion for Cardiomyocytes Isolation

The hearts were rapidly excised from rats anesthetized with sodium pentobarbital (40 mg/kg, i.p.) as described above. Immediately after removal, spontaneously beating hearts were rinsed by immersion in ice-cold Myocyte Isolation Buffer (MIB) containing 120 nmol/L NaCl, 5 mmol/L KCl, 2 mmol/L NaAc, 2 mmol/L MgCl_2_, 1 mmol/L Na_2_HPO_4_, 20 mmol/L NaHCO_3_, 5 mmol/L glucose, 9 mmol/L taurine, and 10 mmol/L CaCl_2_, pH 7.4. Next, hearts were suspended on a blunt end needle of a Langendorff system with the aorta and maintained at 37 °C. Hearts were perfused in a water-jacketed chamber of the Langendorff mode at a constant flow of 10 mL/min with MIB buffer containing 1 mmol/L CaCl_2_, pH 7.4, at 37 °C, and gassed continuously with 5% carbogen for 5 min.

### 4.3. Isolation of Ventricular Cardiomyocytes

After 5 min of heart perfusion in a Langendorff system with MIB buffer containing 1 mmol/L CaCl_2_, the buffer was replaced with MIB buffer containing 5 µmol/L CaCl_2_. Then, hearts were perfused for another 5 min. Low CaCl_2_ level induced a loss of cardiomyocytes contractility. Mild myocardial swelling was obtained using 4-(2-hydroxyethyl)-1-piperazineethanesulfonic acid (HEPES) buffer (120 mmol/L NaCl, 5 mmol/L KCl, 2 mmol/L MgCl_2_, 5 mmol/L glucose, 9 mmol/L taurine, 5 mmol/L HEPES) containing 40 µmol/L CaCl_2_, 25 mg of collagenase, and 2 mg of protease at pH 7.4. Then, the right ventricle was excised from the heart, rinsed with HEPES buffer containing 100 µmol/L CaCl_2_, 150 mg bovine serum albumin (BSA), and minced thoroughly in the digestion solution (HEPES buffer containing 100 µmol/L CaCl_2_, 150 mg BSA, 15 mg collagenase and 1 mg protease). The minced tissue was digested up to six times for 10 min in a water bath [37 °C]), and the third–sixth fraction were used for further experiments [15].

### 4.4. Chemical Ischemia of Isolated Ventricular Cardiomyocytes

**Figure 10:**
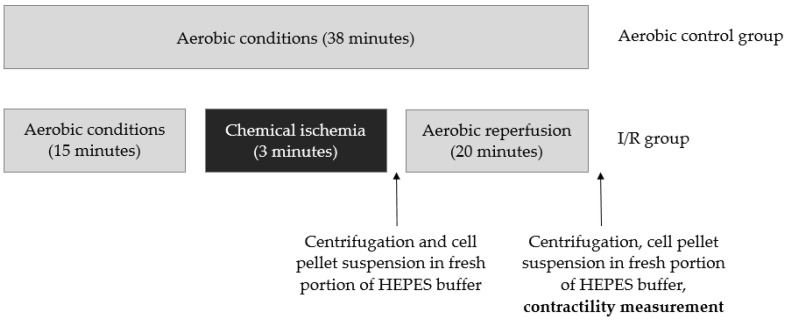
Experimental protocol for chemical ischemia of isolated ventricular cardiomyocytes and aerobic control group.

After 15 min of treatment with HEPES buffer containing 100 µmol/L CaCl_2_ and 150 mg BSA, chemical ischemia was induced by resuspending cells pellets in HEPES buffer containing 4 mmol/L 2-deoxyglucose and 40 mmol/L sodium cyanide. The optimal duration of ischemia—3 min—was established in earlier studies [7]. Three minutes ischemia resulted in an approximately 50% loss of cells contractility, while cells viability remained at 70% or higher. After 3 min of incubation, the buffer containing 2-deoxyglucose sodium cyanide was removed by centrifugation (1 min 1500× *g*); next, the cells pellet was suspended in the fresh portion of HEPES buffer containing 100 µmol/L CaCl_2_ and 150 mg BSA. After reperfusion (20 min), cells were centrifuged (5 min 1500× *g*), and the cells pellet was suspended in HEPES buffer (100 µmol/L CaCl_2_ and 150 mg BSA) and used for contractility measurement. The aerobic control group was kept for 38 min in atmospheric air (Figure 10).

### 4.5. Measurement of Ventricular Cardiomyocytes Contractility

A 100 µl aliquot of cell suspension was placed in the rapid change stimulation chamber of the IonOptix Contractility System (IonOptix, Milton, MA, USA) for 3 min of aerobic stabilization, followed by perfusion using oxygenated HEPES buffer containing 2 mmol/L CaCl_2_ (4 mL/min) at 37 °C. Cardiomiocytes were continuously paced with 1 Hz and 5 V (IonOptix MyoPacer, Milton, MA, USA). The contractility, which was expressed as percentage of peak shortening in comparison to the length of the diastolic cell, was measured on an average of five cells per aliquot. At least five aliquots per heart were examined A total of 13 hearts were tested in each group [15].

### 4.6. Preparation of Heart Homogenates

Hearts previously frozen at −80 °C were crushed with use of a mortar and pestle in liquid nitrogen. Then, hearts underwent three cycles of freezing (in liquid nitrogen) and thawing (at 37 °C) in the homogenization buffer containing 50 mmol/L Tris-HCl (pH 7.4), 3.1 mmol/L sucrose, 1 mmol/L dithiothreitol, 10 mg/mL leupeptin, 10 mg/mL soybean trypsin inhibitor, 2 mg/mL aprotinin, and 0.1% Triton X-100. Homogenates were centrifuged at 10,000× *g* at 4 °C for 15 min. Supernates were collected and stored at −80 °C.

### 4.7. Measurement of Protein Concentration

Protein concentration in cardiac tissue homogenates was determined using Bradford Protein Assay (Bio-Rad, Munich, Germany) and BSA (heat shock fraction, ≥98%, Sigma-Aldrich, Saint Louis, MO, USA) which served as a protein standard.

### 4.8. Analysis of LDH Activity

A lactate dehydrogenase activity assay kit (Sigma-Aldrich, Saint Louis, MO, USA) was used to determine LDH activity in coronary effluents collected during reperfusion, following the manufacturer’s instruction. Briefly, lactate dehydrogenase catalyzes the interconversion of pyruviate and lactate with the reduction of NAD to NADH, which was specifically detected with a colorimetric assay at 450 nm. Lactate dehydrogenase served as a marker of tissue damage. It is naturally located in the cytoplasm and immediately released into extracellular space during cell membrane damage/increased permeability.

### 4.9. Assessment of Oxidative Stress in Rat Hearts

The total ROS/RNS in the rat heart tissue was assessed using an OxiSelect™ In Vitro ROS/RNS Assay Kit (Cell Biolabs, San Diego, CA, USA). The assay measures total ROS and RNS, including hydrogen peroxide, nitric oxide, peroxyl radical, and peroxynitrite anion, using a proprietary fluorogenic probe, dichlorodihydrofluorescin DiOxyQ (DCFH-DiOxyQ). The probe is primed with a dequenching reagent to the highly reactive DCFH form. In the presence of ROS and RNS, the DCFH is rapidly oxidized to the highly fluorescent DCF. Fluorescence intensity is proportional to the total ROS/RNS level in the sample. The total ROS/RNS level was assessed in coronary effluents and normalized to CF [43].

### 4.10. Analysis of MMP-2 Concentration in Heart Homogenates

MMP-2 content in heart homogenates was measured using quantitative Quantikine ELISA Assay for Total MMP-2 (R&D Systems, Minneapolis, MN, USA) according to the manufacturer’s instructions. Total MMP-2 Quantikine ELISA Assay recognized recombinant MMP-2, natural human, mouse, rat, porcine and canine active, pro- and TIMP complexed MMP-2. MMP-2 immobilized with monoclonal antibody specific to this protein was detected with use of anti-Total-MMP-2 polyclonal antibody conjugated to Streptavidin-Horse Radish Peroxidase (HRP). Next, 3,3′,5,5′-Tetramethylbenzidine (TMB) substrate solution was added to develop the reaction. The minimum detectable concentration was 0.033 ng/mL. MMP-2 concentration in heart homogenates was expressed as ng per µg of total protein.

### 4.11. Determination of MMP-2 Activity

MMP-2 activity in heart homogenates was analyzed using the gelatin zymography method with Heussen and Dowdle protocol modified by us [44,45]. Initially, the protein content in heart homogenates was measured as previously described. Heart homogenates were adjusted to the same protein concentration with distilled water and mixed with 4x Laemmli Sample Buffer (Bio-Rad Laboratories, Hercules, CA, USA) in a 4:1 ratio (*ν:ν*). Samples containing 120 µg of protein were applied to 8% polyacrylamide gel copolymerized with gelatin (2 mg/mL) and 0.1% SDS (denaturing, but not reducing conditions). After electrophoresis, gels were rinsed three times for 20 min in 2.5% Triton X-100 to remove SDS. Then, gels were placed in incubation buffer (50 mol/L Tris-HCl pH 7.5, 5 mmol/L CaCl_2_, 200 mmol/L NaCl, 0.05% NaN_3_) and incubated overnight at 37 °C. After gelatin was digested, gels were stained in staining solution (0.5% Coomassie Brilliant Blue R-250, 30% methanol, 10% acetic acid) for 2 h and destained in destaining solution (30% methanol, 10% acetic acid) until bands were clearly visible. MMP-2 activity was visualized as bright bands on a dark background. Zymograms were scanned using a GS-800 Calibrated Densitometer (model PowerLook 2100 XL-USB) and analyzed using Quantity One v. 4.6.9 software (Bio-Rad Laboratories, Hercules, CA, USA). The relative activity of MMP-2 was established and expressed in arbitrary units (AU) as an activity per µg of total protein.

### 4.12. MYL4 and MYL3 mRNA Expression

Total RNA was isolated from heart tissue using TRIZOL Reagent (ThermoFisher Scientific, Waltham, MA, USA) in accordance with the manufacturer’s instruction. Reverse transcription of 2 µg of pure RNA to cDNA using iScript cDNA Synthesis Kit (Bio-Rad, Hercules, CA, USA) was performed. The expression of *MYL4* (coding ALC1) and *MYL3* (coding VLC1) genes in relation to the *GAPDH* gene was analyzed by relative RQ-PCR using CFX96 Touch (Bio-Rad, Hercules, CA, USA). iTaq Universal Sybr Green Supermix (Bio-Rad, Hercules, CA, USA), forward and reverse primers (final concentration 0.1 µmol/L), Ultra Pure DEPC-Treated water and cDNA (100 ng) were used in a final volume of 30 µL. The 5′-3′ sequences of primers were as follows: *MYL4* F: AGGTGGAGCAGCTGTTGACT, *MYL4* R: GAGACTCCACACTGGGCTTC, *MYL3* F: GCTGAGCCTCTCAGGAAGC, *MYL3* R: GACAGAAAGGGTACCACGGG, *GAPDH* F: AGTGCCAGCCTCGTCTCATA, *GAPDH* R: GATGGTGATGGGTTTCCCGT. The amount of mRNA in relation to *GAPDH* was calculated as 2^−ΔCt^. The relative expression of respective genes was compared in hearts exposed to aerobic conditions and hearts subjected to global ischemia.

### 4.13. Analysis of ALC1 and VLC1 Content in Heart Homogenates and Coronary Effluents

ALC1 and VLC1 content in heart homogenates and coronary effluents were determined using a Rat Myosin Light Chain 4 (MYL4) ELISA Kit (sensitivity: 0.1 ng/mL; MyBioSource, San Diego, California, United States of America) and Rat Myosin Light Chain 3 ELISA Kit (sensitivity: 6.99 × 10^−3^ ng/mL; Bioassay Technology Laboratory, Shanghai, China). Briefly, capture antibodies bound ALC1/VLC1 from heart homogenates/coronary effluents. Next, antigens were detected by biotinylated antibodies. HRP conjugated with biotinylated ALC1/VLC1 antibody. TMB substrate solution was added to enable complex visualization. Concentrations of ALC1/VLC1 in heart homogenates were expressed as ng per µg of total protein and as ng/mL normalized to coronary flow in coronary effluents.

### 4.14. Statistical Analysis

GraphPad Prism v. 6 was used for statistical analysis of the results. A Shapiro–Wilk normality test or Kolmogorov–Smirnov test was used to assess the normality of variances changes. Next, results were analyzed by appropriate tests: paired *t* test, unpaired *t* test, and Mann–Whitney U-test. Correlations were estimated using Pearson’s or Spearman’s tests. Results were expressed as mean ± SEM. *p* < 0.05 was accepted as a statistically significant difference.

## 5. Conclusions

In conclusion, oxidative stress caused the increase in MMP-2 activity, which led to cell damage visible in substantially decreased hemodynamic parameters and VLC1 expression changes. Oxidative stress also induced ALC1 gene expression and synthesis in hearts, which may constitute an adaptive mechanism to I/R conditions. This study might also be a good foundation for further research into actin–myosin interactions in I/R conditions.

## Figures and Tables

**Table 1:** Effect of ischemia/reperfusion injury on heart rate, left ventricular developed pressure, and coronary flow of isolated rat hearts.

Parameter	Groups		*p*-Value ^d^
	Aerobic Group *n* = 9	I/R Group *n* = 7	
HR (bpm) ^a^	302.1 ± 17.6	51.9 ± 34.6	0.0003
LVDP (mm Hg) ^a^	51.9 ± 5.8	18.4 ± 11.9	0.0549
CF (mL/min) ^b^	14.1 ± 1.1	7.9 ± 2.2	0.0175
Recovery (%) ^c^	92.6 ± 3.4	21.3 ± 14.1	0.0012

Mean ± SEM; ^a^ After ischemia/reperfusion (I/R) (77th minute of the experiment); ^b^ After ischemia (47th minute–first minute of reperfusion); ^c^ Difference between recovery in 25th and 77th minutes of experiment; ^d^ Mann–Whitney U test/Unpaired *t*-test.

## Abbreviations

Aero	Aerobic control group
ALC1	Atrial light chain 1
AU	Arbitrary units
BSA	Bovine serum albumin
CF	Coronary flow
DCF EDTA	2′,7′–dichlorodihydrofluorescein ethylenediaminetetraacetic acid
ELC	Essential light chain
FLC1	Fetal light chain 1
GAPDH	glyceraldehyde 3-phosphate dehydrogenase
HEPES	4-(2-hydroxyethyl)-1-piperazineethanesulfonic acid
HR	Heart rate
HRP	Streptavidin-Horse Radish Peroxidase
I/R	Ischemia/reperfusion
IRI	Ischemia/reperfusion injury
LDH	Lactate dehydrogenase
LVDP	Left ventricular developed pressure
MHC-α	Myosin heavy chain α
MHC-β	Myosin heavy chain β
MIB	Myocyte isolation buffer
MLCs	Myosin light chains
MLCs1	Myosin light chains 1
MLCs2	Myosin light chains 2
MMP-2	Matrix metalloproteinase 2
*MYL3*	Gene coding ventricular light chain1
*MYL4*	Gene coding atrial light chain1
RLC	Regulatory light chains
ROS/RNS	Reactive oxygen/nitrogen species
RPP	Recovery of rate pressure product
TMB	3,3′,5,5′-Tetramethylbenzidine
VLC1	Ventricular light chain 1
